# Impact of Metal Ions on Cellular Functions: A Focus on Mesenchymal Stem/Stromal Cell Differentiation

**DOI:** 10.3390/ijms251810127

**Published:** 2024-09-20

**Authors:** Kirsten Peters, Susanne Staehlke, Henrike Rebl, Anika Jonitz-Heincke, Olga Hahn

**Affiliations:** 1 Institute of Cell Biology, Rostock University Medical Center Rostock, Schillingallee 69, 18057 Rostock, Germany; susanne.staehlke@med.uni-rostock.de (S.S.); henrike.rebl@med.uni-rostock.de (H.R.); olga.hahn@med.uni-rostock.de (O.H.); 2 Research Laboratory for Biomechanics and Implant Technology, Department of Orthopaedics, Rostock University Medical Center, Doberaner Strasse 142, 18057 Rostock, Germany; anika.jonitz-heincke@med.uni-rostock.de

**Keywords:** mesenchymal stem/stromal cells (MSCs), metal ions, metalloproteins, cell differentiation, oxidative stress, inflammation, cell signaling, cell adhesion, cell migration, tissue regeneration

## Abstract

Metals play a crucial role in the human body, especially as ions in metalloproteins. Essential metals, such as calcium, iron, and zinc are crucial for various physiological functions, but their interactions within biological networks are complex and not fully understood. Mesenchymal stem/stromal cells (MSCs) are essential for tissue regeneration due to their ability to differentiate into various cell types. This review article addresses the effects of physiological and unphysiological, but not directly toxic, metal ion concentrations, particularly concerning MSCs. Overloading or unbalancing of metal ion concentrations can significantly impair the function and differentiation capacity of MSCs. In addition, excessive or unbalanced metal ion concentrations can lead to oxidative stress, which can affect viability or inflammation. Data on the effects of metal ions on MSC differentiation are limited and often contradictory. Future research should, therefore, aim to clarify the mechanisms by which metal ions affect MSC differentiation, focusing on aspects such as metal ion interactions, ion concentrations, exposure duration, and other environmental conditions. Understanding these interactions could ultimately improve the design of biomaterials and implants to promote MSC-mediated tissue regeneration. It could also lead to the development of innovative therapeutic strategies in regenerative medicine.

## 1. Introduction

Metals play a decisive role in the human body at various levels. Most metals are bound to proteins (metalloproteins), and about half of all proteins are assumed to contain a metal [1]. The form of the protein-bound metals is always ionic, but the oxidation state can vary. Due to the high complexity of metal–protein interactions, it must be assumed that the effects of the availability of metals on biological networks still need to be sufficiently investigated [1].

Around ten metals are essential for life, including sodium (Na), potassium (K), magnesium (Mg), calcium (Ca), manganese (Mn), iron (Fe), cobalt (Co), zinc (Zn), nickel (Ni), copper (Cu), and molybdenum (Mo) [2]. The biologically essential metals can be divided into two groups: non-transition elements (e.g., Na, K, Mg, and Ca) and transition elements (e.g., Fe, Co, and Cu). Non-transition elements have a relatively constant oxidation state, and their ions have completely filled electron shells. In contrast, transition elements have variable oxidation states, and their ions have incompletely filled electron shells. Incompletely filled electron shells manifest unique physical and chemical properties (absorption bands in the ultraviolet and visible spectral range, paramagnetism, etc.) [2].

The essential metal ions include ions, such as divalent Ca^2+^, which are present in comparatively large quantities: Ca^2+^ makes up around 1–2% of the human body weight and is an essential component of bones and teeth [3]. Mg^2+^ is also an important element in comparatively large quantities in the human body (around 0.05% of body weight) [4]. In addition to these essential, non-transition elements present in large quantities, the essential transition metals are in smaller quantities. Transition metals whose storage and transport are particularly important are Fe, Zn, Cu, Mo, Mn, Co, vanadium (V), and Ni [5]. Fe, for example, makes up only a negligible proportion of our body weight (around 1 to 3 g for an adult) but is essential for many physiological functions [6]. The same applies to the amount of Zn, which is estimated to be around 2 g in adults. Around 60% of the total Zn content is found in skeletal muscle and around 30% in bone mass. Plasma Zn accounts for only about 0.1% of the total zinc content in the body but is subject to strict homeostatic control [7]. However, there is still debate about which metals are essential or beneficial for a particular organism and which are non-essential and only toxic [8,9], such as chromium (Cr^3+^), where results from laboratory animals indicated the essentiality. However, recently, it became clear that Cr^3+^ is not essential for humans [10] but appears to affect iron absorption [11], whereas Cr^6+^ is considered toxic as it may be involved in the development of chromium-induced cancer [12]. A significant amount of non-essential metal ions, such as lithium (Li), platinum (Pt), rubidium (Rb), strontium (Sr), zirconium (Zr), but also gold (Au), and uranium (U) are found in the human body due to artificial exposure through food, air, or metallic biomaterials [13]. Due to their chemical similarity to important essential metals, there is often an affinity for the same donor atoms and chemical sites of the essential metals. For example, Li^+^ (applied as a drug for the treatment of bipolar disorder) [14] and Rb^+^ are shown to replace Na^+^ [15]. Ultimately, the uptake of an element by a biological system and its use in biochemical processes is no proof of essentiality [9].

**Figure 1:**
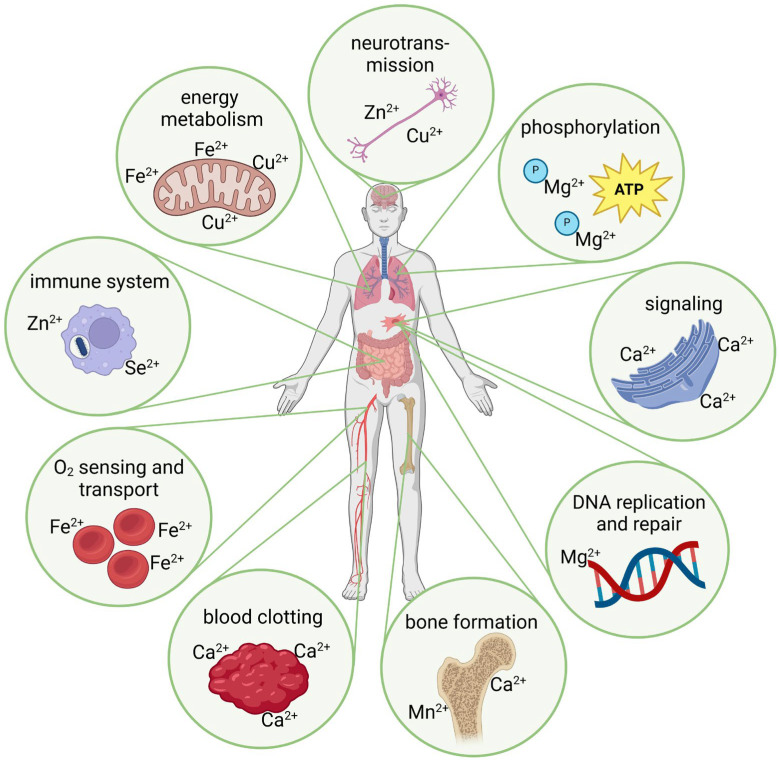
Schematic depiction of exemplary metal ion-induced effects in physiological processes (created with BioRender.com, agreement No. DM274QKXG3).

Metal ions often act as cofactors for enzymes and are essential for their functioning, enabling their catalytic activity. Metal ions are also responsible for the structural stability of proteins and regulating numerous biochemical reactions (Figure 1). The binding of a non-specific metal ion, i.e., an ion that does not have a specific binding capacity, can distort the geometry of a metal binding site and, thus, impair the activity of the respective metalloprotein. The biological challenge is, therefore, to prevent the tight association of non-specific metal ions in proteins [16]. As mentioned, transition metal ions have different oxidation states depending on the elements they combine with and the specific conditions. The oxidation state of metal ions is decisive for their chemical and, therefore, also their biological characteristics [17]. Fe, for example, has a remarkable variety of oxidation states (between −2 and +6), which strongly influence its chemical properties and biological effectiveness. The most common and biologically relevant oxidation states are +2 and +3. The human body more readily absorbs Fe^2+^, which plays an important role in hemoglobin for oxygen transport. Fe^3+^ is less soluble, more challenging to absorb, and often found in Fe storage proteins, such as ferritin. The ability of Fe to switch between different oxidation states is crucial for many biological processes, especially for redox reactions in enzymes. For example, switching between Fe^2+^ and Fe^3+^ enables electron transport in the respiratory chain. Controlling the oxidation state of iron is also essential to prevent oxidative stress [18,19]. Free Fe^2+^ can form harmful free radicals (Section 2.3), which is why the organism carefully regulates and stores iron [20]. Due to metal ions’ high reactivity and catalytic potential, their content in our diet and our environment is an essential factor for health and the development and progression of diseases. Even small shifts in the ionic balance have an impact. A better understanding of metals’ mode of action in biology could answer many unsolved medical, biochemical, and biological problems [8].

Some critical aspects of the role of metal ions in physiological processes include oxygen transport (i.e., primarily Fe^2+^ in hemoglobin and myoglobin), phosphorylation of molecules (e.g., Mg^2+^ is a cofactor for many enzymes, which, among other things, allows the storage of high-energy phosphates, such as in adenosine triphosphate/ATP), electron transport (e.g., copper ions (Cu^2+^) and Fe^2+^/Fe^3+^ involved in electron transport chains, particularly in the mitochondria), and DNA replication and repair (e.g., Zn^2+^ and Mg^2+^) (Figure 1). Furthermore, cell signaling is performed in the presence of Ca^2+^ as a second messenger in cell signaling (Section 2.2), Cu^2+^ and Zn^2+^ are involved in neurotransmitter synthesis, Zn^2+^ and selenium ions (Se^2+^) in the development and activity of immune cells, and Cu^2+^ and Mn^2+^ are components of the antioxidant enzymes, which help to scavenge reactive oxygen species (ROS) (Section 2.3). Although these metal ions are essential for physiological processes, a deficiency, excess, or imbalance in their concentrations can lead to significant molecular deviations and, thus, have the potential to cause various disorders [21].

The toxic effects of an excess of metal ions have already been described many times. The adverse impacts of metal ions can vary based on the specific metal (this applies to heavy metal ions), its chemical form or oxidation state, and its concentration [17]. Prolonged exposure to metal ions often correlates with heightened mutagenicity and an elevated risk of cancer, exemplified by Cr^6+^ [22], Ni^2+^ [23], or heavy metal ions, such as ions from cadmium (Cd^2+^) [12]. The development of ROS and, thus, oxidative stress stemming from an unbalanced or physiological metal ion exposure appears to be the focal point of exerted toxicity. Given the extensive documentation of these severe toxic effects, this review article will only delve into them briefly. In addition, this review article does not address the complex area of allergies to metals, such as nickel, cobalt, or chromium [24,25]. Instead, we will focus on attenuated effects that affect physiological responses at the cellular levels, emphasizing mesenchymal stem/stromal cells (MSCs), also known as mesenchymal progenitor cells.

MSCs are widely distributed throughout the various tissues of the body [26]. They exhibit a versatile ability to differentiate in multiple directions and play a crucial role in maintaining tissue homeostasis, facilitating repair, and modulating the immune response. The differentiation capacity of MSCs allows them to develop into cells of the mesodermal lineage, such as adipocytes, osteocytes, or chondrocytes [27]. They are also reported to differentiate into neuron-like cells. Notably, MSCs from different sources exhibit variations in surface marker expression and differentiation potential. These cells are now being used clinically to treat the consequences of autoimmune diseases, graft-versus-host diseases, and sepsis-related complications, among other diseases [28]. As MSCs are a crucial cell type in the tissue regeneration processes, it is essential to increase knowledge about the effects of metal ions on their behavior. Therefore, our review addresses the effects of physiologically and incidentally supplied metal ions. It has been shown that metal ions can modulate the differentiation potential of MSCs, but there are still many unanswered questions. Understanding the interaction of metals and MSCs not only has the potential to allow re-evaluation of metal ion supplementation and the use of metals in the body but also opens the way for innovative strategies in regenerative medicine. As the interactions between metal ions and MSCs have not yet been sufficiently explored, this review also addresses the effects of metal ions on the functions and differentiation of other relevant cell types to provide a more comprehensive current picture on this topic. To this end, relevant cell functions are first described in general terms in the individual sections and then discussed concerning MSCs when specific information is available.

## 2. Biological Metal Ion Interaction

### 2.1. Mechanisms of Metal Ion Uptake

All organisms require mechanisms to strictly control metal levels to ensure sufficient but non-toxic amounts of metal ions are available. The uptake of metal ions into cells is mainly mediated by specialized transport proteins and ion channels in the cell membrane [29]. These proteins can recognize and bind to specific metal ions, allowing them to enter the cell by passive diffusion, facilitated diffusion, or active transport, depending on the type of ion and the concentration gradient [30].

In passive diffusion, small metal ions, such as Cu^2+^ and Zn^2+^, can diffuse through membranes when concentrations are higher outside the cell, mainly when they are unbound or in a non-complexed form. This process requires no energy and is influenced by the size and charge of the ions [29,30]. During facilitated diffusion, certain metal ions utilize specific transport proteins that facilitate their movement through the membrane without energy expenditure. For example, specific ion channels allow certain metal ions to enter the cell passively when the extracellular concentration is higher. Metal ions, as described for ions, such as Ca^2+^, Mg^2+^, and Zn, are actively transported into the cells against their concentration gradient [31]. This energy-dependent process involves specialized ion channels and transporters, which play a central role in maintaining precise intracellular ion concentrations [29,30]. Iron, for example, is introduced into the cells via an active, energy-intensive process, endocytosis, which is bound to the circulating iron transport protein transferrin [32]. Once inside the cell, the metal ions are crucial for various cellular processes, including signaling pathways, differentiation, and maintaining cellular homeostasis.

Metallic nanoparticles (with a size of less than 100 nm by definition) penetrate the cells relatively similarly to metal ions. Depending on their size, nanoparticles have an immense surface area per unit volume and, therefore, a high proportion of atoms in the layers close to the surface and the ability to exhibit quantum effects [33,34,35]. Due to their high reactivity and very high ability to penetrate cells, metallic nanoparticles are of great interest for research and diagnostic and therapeutic medical technology [33,35]. However, due to the exceptional properties of nanoparticles, we have only looked at them marginally in this review, even though we are aware of their considerable potential but also their risks.

### 2.2. Calcium Signaling

With a weight up to approximately 1.2 kg, calcium is the most abundant metal in the human organism [36]. In the body, 99% of the calcium is bound in bones and teeth as hydroxyapatite (Ca_5_(PO_4_)_3_(OH)), providing mechanical stability and strength [2,3,37]. Only 0.1% of calcium is found in the extracellular fluid, and approximately 50% of this is present in a freely ionized and, thus, biologically active form as the divalent Ca^2+^ ion [38,39]. The regulation of the Ca^2+^ concentration in blood plasma to the standard value of 2.45 mM, calcium homeostasis, is a complex process that takes place in the intestine, kidneys, and bones under the control of, e.g., parathyroid hormone (PTH), calcitonin, and 1,25-dihydroxyvitamin D [40,41,42]. The bones serve as a calcium store, and part of Ca^2+^ is released via hormonal regulation in the case of calcium deficiency [43,44,45]. In addition to the stabilizing and storing function, Ca^2+^ supports various physiological tasks, such as muscle contraction [40,46], blood clotting [4,40], and the transmission of nerve impulses [47,48].

In cells, Ca^2+^ acts as a ubiquitous second messenger crucial for regulating various cellular signal transduction processes [49,50,51]. In this function, Ca^2+^ impacts, e.g., cell adhesion [52,53], enzyme activation and activity [40], cytoskeletal organization [54,55], cell proliferation [56,57], and differentiation [58,59,60]. The basal cytoplasmic Ca^2+^ concentration is tightly regulated within narrow limits (~100 nM) by intracellular calcium stores (i.e., the endoplasmic reticulum or the mitochondria), membrane channels and pumps, and intracellular calcium-binding proteins [31,42]. For Ca^2+^ to act as a second messenger, the intracellular concentration must increase briefly due to a specific stimulus [40,51]. Ca^2+^ can be released from the extracellular space via calcium channels in the plasma membrane [58,61]) or intracellularly via different receptors in the endoplasmic reticulum [2,38,51,62]. The Ca^2+^ increase induced in this way can be up to ~1000 nM and only occurs briefly [31,42]. Therefore, calcium pumps and exchangers ensure the reverse to regulate intracellular Ca^2+^ concentration [58,62,63]. Calcium sensing/binding proteins, such as calmodulin, which bind Ca^2+^ intracellularly and subsequently initiate a downstream signaling cascade through conformational changes, are essential in the regulatory and signaling pathways [2,64]. This tight regulation of calcium signaling in cells is highly complex and essential for the proper functioning of cells [36,38,50].

The binding of non-specific metal ions can modulate intracellular calcium signaling through various mechanisms, such as altering the expression or activity of key components, as they compete directly with Ca^2+^ for binding sites on calcium sensor proteins and ion channels [2,51,65]. Some ionic radii are close to those of Ca^2+^ (i.e., 0.99 Å), e.g., Mn^2+^ (0.8 Å) and Fe^3+^ (0.76 Å) [2]. The binding of other metal ions, such as Zn^2+^, Ni^2+^, and Mg^2+^, to binding sites of calcium channels/transporters/pumps can either modulate their gating properties or block their activity, thereby affecting calcium influx and intracellular calcium dynamics [2,36,38,66]. Thus, Mg^2+^ competes for the most Ca^2+^ binding sites and acts as a calcium antagonist [2,67]. Moreover, the Ca^2+^-ATPase pump must distinguish between Ca^2+^ and the other metal cations (such as Na^+^, K^+^, and Mg^2+^) [2]. Furthermore, metal ions can trigger Ca^2+^ release from the endoplasmic reticulum by modulating the corresponding calcium channels [58,68]. In addition, the binding of Mg^2+^ or the heavy metal ion Cd^2+^ to the calcium-binding sensor protein calmodulin also leads to amended signaling [69].

Extracellular metal ions could favor Ca^2+^ influx through voltage-activated Ca^2+^ channels, which promote osteogenic differentiation [70,71,72]. Extracellular Ca^2+^ is essential in bone remodeling as it influences calcium-sensing receptors in osteoblastic cells and induces corresponding signaling pathways [73]. Furthermore, appropriate Ca^2+^ and related PO_4_^3−^ concentrations positively affect cell proliferation, mineralization, and osteogenic differentiation of MSCs [72,74]. Also, Mg^2+^ competes for calcium channels, potentially promoting bone development [75]. Other metal ions, such as Zn^2+^, can activate calcium-dependent signaling pathways in MSCs that regulate their differentiation, especially towards the osteogenic lineage [76,77]. Sr^2+^, for example, has ionic characteristics similar to Ca^2+^, and it can bind to phosphates in human bones more significantly than many essential metals. Although Sr^2+^ is not essential, it has been shown to protect against osteoporosis, and its ranelate salt has been categorized as a useful drug [78]. Other metal ions, such as Co^2+^, act as calcium agonists and, thus, stimulate Ca^2+^ release by finally activating the canonical Wnt/β-catenin pathway, an essential pathway in osteogenic differentiation [79]. For this reason, materials (e.g., bioactive glasses) containing rare ions are currently being developed and characterized to influence the biological effects in the implant periphery [80] (Section 2.7).

It is described that excessive amounts of iron ions cause oxidative stress and increased calcium signaling (e.g., by Ca^2+^/calmodulin-dependent protein kinase β), which can ultimately lead to cell death. Iron overload, which can occur in various chronic diseases, such as hereditary hemochromatosis or diabetes, leads to limitations in the normal functionality of MSCs. The impaired functionality is due to changes in many signaling pathways involved in cell survival, proliferation, and differentiation (including calcium signaling and oxidative stress) [81]. However, even persistently high Ca^2+^ concentrations (>10 mM) have a cytotoxic effect [70], and uncontrolled Ca^2+^ release overwhelms the buffering capacity of the cell, leading to abnormal/defective activation of calcium-dependent signaling pathways [40,58].

In summary, the influence of non-specific metal ions on intracellular calcium signaling pathways is a critical issue that requires further investigation. Most studies have focused on the effects of single metal ions. However, the combined or synergistic effects of multiple metal ions on calcium signaling and MSC differentiation are still less well understood. Most existing studies have been performed in vitro, and further, in vivo studies are needed to validate the therapeutic potential and the risks of metal ion-containing biomaterials for regenerative therapies [82]. Understanding the underlying mechanisms by which these metal ions disrupt calcium homeostasis and signaling could provide valuable insights into developing novel therapeutic strategies to mitigate the adverse health effects of metal ion exposure and toxicity.

### 2.3. Influence of Metal Ions on the Redox Balance and the Formation of Reactive Oxygen and Nitrogen Species

ROS and reactive nitrogen species (RNS) are highly reactive molecules that contain oxygen and nitrogen, respectively. ROS and RNS play a dual role in the body: they are essential signaling molecules and cause cell damage if their concentrations exceed the body’s ability to decompose or repair the resulting damage. This detrimental condition is known as oxidative stress and can cause damage to cellular components, such as lipids, proteins, and DNA, contributing to various diseases or the aging processes [83]. Generally, the generation of ROS is localized, particularly in the mitochondrial respiratory chain, where 85% of oxygen is metabolized, and a small amount of partially reduced oxygen intermediates are generated [84]. The Fenton and Haber–Weiss reactions are the main mechanisms of metal ion-induced ROS formation. In Fenton reactions, the transition metal ion (e.g., Fe^2+^) reacts with hydrogen peroxide (H_2_O_2_) to produce the highly reactive hydroxyl radical (HO^•^) and an oxidized metal ion. In a Haber–Weiss reaction, the oxidized metal ion can then be reduced by superoxide (O2^•−^) in a Haber–Weiss reaction, generating another hydroxyl radical and oxygen [84,85]. Dysfunction of the homeostasis of redox-active metal ions, like Fe^2+^ or Co^2+^, causes them to become available as a catalyst, leading to excessive ROS and RNS formation [86,87,88]. To emphasize the importance of the toxic reaction caused by excessive iron accumulation in the cell, the Fenton reaction leads to an overproduction of ROS, which promotes lipid peroxidation and, thus, a non-apoptotic cell death, ferroptosis [89,90].

RNS include nitrogen dioxide (NO_2_), nitric oxide (NO), and nitrous acid (HNO_2_). For instance, NO is released in the cell after L-arginine is converted into L-citrulline by nitric oxide synthases [91]. The generated NO can further interact with ROS to produce numerous RNS involved in oxidative and nitrosative damage [92]. However, besides its function as an important signaling molecules, RNS can also regulate the activity of metalloenzymes that contain catalytic iron–sulfur clusters [91].

While the generation of ROS and RNS by endogenous sources is mainly related to normal metabolism or immune cell functions, exogenous sources, especially metals, directly stimulate ROS and RNS and can, thus, also have toxic or carcinogenic effects [93,94]. Apart from the uptake of metals and, thus, metal ions via the environment, several medical procedures could also contribute to increased levels of metal ions in the biological system [85,94,95]. Studies investigating the response of particles and ions to ROS in MSCs provide insights into the complex interaction between these components. Indeed, exposure to different particles and ions can lead to the generation of ROS in MSCs and affect their behavior and function. Specifically, cellular damage and inflammation in response to metal-induced ROS generation have been described for corrosion products from cobalt–chromium or titanium alloys [96]. It is known that cellular senescence is also triggered by prolonged oxidative stress [97]. Against this background, exposure to metals and their corrosion products also harbors a fundamental risk of developing senescence, also in MSCs [98]. Exposure to metallic nanoparticles can also lead to the formation of ROS in MSCs and can affect cell behavior and function. Such induced ROS formation can trigger various deleterious events, including inflammation, fibrosis, genotoxicity, and carcinogenesis, and is influenced by different physicochemical properties [85]. For example, zinc oxide nanoparticle-induced apoptosis of MSCs occurs when lysosomal degradation destabilizes the nanoparticles, releasing large amounts of Zn ions and forming ROS [99,100].

The cell has several enzymatic and non-enzymatic systems to neutralize the harmful effects of the ROS produced via excessive metal ion exposure. Enzymatic antioxidants primarily include catalase (CAT), glutathione peroxidase, thioredoxin, and superoxide dismutase (SOD) [92,101]. Paradoxically, SOD and CAT contain metal ions as an integral part of their active centers to counteract the toxic effects of free radicals [93]. Thus, the toxicity of redox-active transition metal ions, such as iron or copper, in the living system is highly complex, as they are integral components of the active protein sites of many antioxidants and, thus, trigger redox cycle reactions [83,102].

SODs are considered the most critical enzymatic antioxidants that catalyze the dismutation of superoxide radicals to hydrogen peroxide and molecular oxygen, while glutathione peroxidase and CAT subsequently cleave the hydrogen peroxide produced by SODs to water. Cells express three types of SODs: the homodimer copper- and zinc-containing SOD (CuZnSOD, SOD1), which is mainly localized in the cytoplasm. Moreover, smaller amounts can also be detected in the intermembrane space of the mitochondria. The extracellular SOD (ECSOD, SOD3), which contains both copper and zinc in its active center, is localized in the extracellular area of the cell. MnSOD (SOD2), instead, is found exclusively in the mitochondrial matrix, where it binds divalent or trivalent Mn or Fe ions as cofactors in the active center [101].

Studies on the influence of SOD activity by non-specific metal ions are rare. For example, there is no information on the direct effects of Co^2+^ on the metal ion-carrying SOD. An early study on the administration of 25 mg/kg CoCl_2_ to rabbits, however, found a significant reduction in SOD activity in erythrocytes [103]. It is assumed that in SOD2, after the substitution of the metal bond by Co^2+^, the conversion to the trivalent cobalt ion occurs, which leads to a redox potential that is outside the range required for the conversion of the superoxide radical to hydrogen peroxide and, thus, explains the inactivity of the enzyme [104]. Since cobalt overload also leads to increased oxidative stress, a reduction in the activity of an enzyme that prevents oxidative stress could have a decisive influence on the further course of the reactions and, thus, related diseases [105].

MSCs play a crucial role in combating oxidative or nitrosative stress through various mechanisms involving antioxidant enzymes, such as SOD or glutathione peroxidase. In addition, MSCs constitutively express heat shock protein 70 (HSP70) and sirtuin 3, which are also thought to play a significant role in the resistance of MSCs to oxidative/nitrosative damage [106]. The balance between ROS production and antioxidant defense mechanisms is crucial for the fate of MSCs, as the regulation of ROS levels ensures the maintenance of MSC function and survival. Excess ROS is associated with aging processes, as the impaired regulation of cell metabolism leads to cell damage or dysfunction [107]. The relationship between age-related loss of function and ROS in stem cells has been the subject of limited research. However, increased ROS content in MSCs leads to a reduction in the expression of SOD, which promotes aging processes [108]. In the same study, additional exposure to extracellular vesicles released from juvenile MSCs led to improved functionality of mature MSCs by decreasing their ROS content [108]. Extracellular vesicles are small, membrane-enclosed structures released by cells into the extracellular environment, contain various biomolecules, and are crucial for intercellular communication. The enhancement of MSC function induced by extracellular vesicles can be attributed, particularly to the high content of antioxidant enzymes in juvenile, non-differentiated MSCs, which are presumably also enriched in their extracellular vesicles [109]. Further studies have shown that either MSCs or MSC-derived extracellular vesicles (in this case, the approximately 100 µm-sized exosomes) can prevent ferroptosis by stabilizing process-relevant proteins, such as glutathione peroxidase or sirtuin [89].

To sum up, the interplay between ROS, antioxidant enzymes, and the cellular redox state is crucial for the function, survival, and therapeutic efficacy of MSCs [106]. Maintaining an optimal ROS balance by modulating SOD and other antioxidants might be decisive for exploiting the full potential of MSC-based therapies.

### 2.4. Inflammation and Immunomodulation

Metal ions can significantly affect inflammation, influencing pro-inflammatory and anti-inflammatory responses through various mechanisms. Metal ions, such as Co^2+^ and Ni^2+^, can induce pro-inflammatory responses by the release of pro-inflammatory cytokines, such as interleukin (IL-)1β, IL-8, monocyte chemoattractant protein-1 (MCP-1) and macrophage colony-stimulating factor (M-CSF), and ROS, in either macrophage [110] or osteoblasts [111], which could also affect the inflammatory environment surrounding MSCs. In addition, Cr ions can form stable chromium phosphates (CrPO_4_) via phosphate compounds, which accumulate in the tissue and cause ROS-related damage in their environment [112].

However, it has also been described that, e.g., Cu^2+^ and Mg^2+^ stimulate the expression of anti-inflammatory markers in a dose-dependent manner. In particular, Mg^2+^ can reduce the expression of pro-inflammatory cytokines, such as IL-6 and tumor necrosis factor (TNF), and reduce the activation of nuclear factor of kappa B (NFκB) in inflammatory (lipopolysaccharide)-activated macrophages [113]. In the same study, it became clear that higher concentrations of Cu^2+^ and Co^2+^ stimulated the expression of pro-inflammatory markers. The relationship between MSCs and metal ions and the associated inflammation is complex. Magnesium, for example, supports the osteogenic differentiation of MSCs and has anti-inflammatory properties that may support MSC function [114,115,116,117,118]. Although MSCs have mechanisms to control iron metabolism, iron overload can impair the differentiation and function of MSCs by promoting the development of ROS and associated oxidative stress [81], and iron chelation can reduce their inflammatory activation [119].

Metal ions can significantly impair the immune system even at low concentrations, and repeated exposure can exacerbate these effects. One example is patients with metal-on-metal hip implants, whose corrosion-intensive metal components can increase the risk of late bacterial infections. In these cases, metallic corrosion products are assumed to contribute to this problem [120]. In this context, cobalt ions have been shown to have an immunosuppressive effect by inhibiting lymphocyte proliferation and cytokine release [121,122]. Further evidence of a suppressed immune response due to metallic corrosion products comes from animal experiments. In these experiments, osteosynthesis materials were deliberately contaminated with *Staphylococcus aureus* during surgery, and the infection rate was about twice as high with highly corrosive stainless steel as with comparatively low-corrosion titanium. The authors conclude that the composition of the metal used in implants influences the immune response in the surrounding tissue [123]. This influence may ultimately lead to poorer clinical outcomes, including an increased risk of septic implant failure [123].

Therefore, metal ions can have diverse and significant effects on inflammation and immune responses, ranging from the promotion of inflammatory responses to the modulation of immune function and anti-inflammatory properties, depending on the specific ion and context. Metal ions play a crucial role in regulating the inflammatory response and the function of MSCs. Understanding these complex interactions is crucial for developing new or improved therapeutic approaches.

### 2.5. Cellular Oxygen Sensing and Hypoxia Signaling

Cellular oxygen sensing encompasses the physiological mechanisms by which cells monitor oxygen levels to maintain their function by adjusting gene expression [124]. Cells have a molecular signaling pathway involving hypoxia-inducible factors (HIFs) that regulate gene expression in response to changes in oxygen levels [125]. At low oxygen levels (termed hypoxia), the HIF proteins are stabilized and translocate to the nucleus, where they activate the expression of genes involved in the adaptive cellular response to hypoxia via the hypoxia-responsive element [126]. Thus, induced hypoxia-adapted gene expression may include increased production of erythropoietin to stimulate red blood cell production, production of the vascular endothelial growth factor (VEGF) to stimulate angiogenesis, as well as altered metabolism to improve oxygen utilization or adapt energy metabolism to a lack of oxygen [127,128,129,130]. HIF signaling is crucial in the cellular adaptation to oxygen availability and control responses that are fundamental for survival and homeostasis. From regulating metabolism and energy production to influencing vascular tone and erythropoiesis, the HIF signaling pathway plays a central role in maintaining tissue function under various oxygen tensions. In addition, dysregulation of the HIF signaling pathway has been identified in several pathological conditions, highlighting its importance beyond normal physiology.

Fe^2+^ plays a crucial role in the recognition and adaptation of cells to changing oxygen partial pressures [131,132]. Fe^2+^ is a cofactor for the prolyl hydroxylase domain (PHD) enzymes, which are essential regulators of HIF signaling [133,134,135]. The binding of Fe^2+^ to the catalytic site of PHDs is crucial for their enzymatic activity. PHD utilizes oxygen and α-ketoglutarate as substrates to hydroxylate specific proline residues on HIF-α subunits under normoxic conditions. This hydroxylation marks HIF-α for recognition by the von Hippel–Lindau (VHL) tumor suppressor protein, which leads to its ubiquitination and subsequent proteasomal degradation [136]. Adequate iron levels consequently ensure the proper function of PHDs and enable the timely degradation of HIF-α subunits in an oxygen-rich environment [131,137]. Thus, clinical iron deficiency interferes with normal responses to hypoxia, which can lead to impaired hypoxia sensing and signaling, providing a mechanism by which iron deficiency may be detrimental to human health [138]. In addition to its role in regulating PHD activity, Fe^2+^ is also involved in other aspects of HIF signaling. For example, Fe^2+^ is required for the activity of FIH (factor inhibiting HIF), another oxygen-sensitive enzyme involved in the hydroxylation of an asparagine residue within the C-terminal transactivation domain of HIF-α. This hydroxylation blocks the interaction between HIF-α and the transcriptional coactivators and, thus, modulates the transcriptional activity of HIF [139,140].

However, it is also known that, in addition to Fe^2+^, other divalent metal ions can influence the stability and, thus, activity of HIF-1 by modulating the activity of prolyl hydroxylases or by interacting directly with HIF-1α. Metal ions known to stabilize HIF-1 include Co^2+^ and Ni^2+^, which can at least partially mimic the effects of hypoxia. These ions compete with iron to bind to the catalytic site of the enzyme, stabilizing HIF-1α even under physiologically normal oxygen concentrations (referred to as normoxia), thus disrupting the physiological oxygen-sensing mechanism [141,142]. In addition, Co^2+^ has been shown to bind directly to the oxygen-dependent degradation domain (ODD) of HIF, possibly interfering with the interaction between HIF and the VHL [143].

Furthermore, Co^2+^ and Ni^2+^ can bind more strongly than Fe^2+^ to specific membrane transporters, potentially suppressing iron release into cells [144]. Thus, induced iron deficiency can impair the function of iron-dependent enzymes such as the PHD. In addition, increased ROS formation, which is particularly confirmed for Cu^2+^, stabilizes HIF-α, either by directly inactivating PHDs [145] or indirectly by degrading ascorbic acid [146], a cofactor necessary for PHD function [93,147].

In summary, divalent metal ions, particularly Fe^2+^, serve as critical cofactors in HIF signaling by promoting the activity of key molecules involved in the oxygen-dependent regulation of HIF-α stability and transcriptional activity. Divalent metal ions, such as Co^2+^, Ni^2+^, and Cu^2+^, significantly affect various physiological oxygen signals that are directly related to cancer, ischemic diseases, and wound healing. The mechanisms of action underlying their effect on HIF-1 regulation may vary depending on the cell type examined, ion concentration, and duration of exposure. Understanding the interplay between metal ion availability and HIF signaling pathways holds promise for elucidating the molecular mechanisms underlying hypoxia-related diseases and may provide opportunities for therapeutic interventions targeting these pathways [124].

#### Energy Metabolism

Metal ions play a crucial role in energy metabolism as they are essential cofactors for many enzymes involved in energy production and cell utilization [148,149]. An important example is iron, which acts as a cofactor for enzymes in the electron transport chain, such as cytochromes, which are crucial for ATP production through oxidative phosphorylation. Magnesium is required for the activity of enzymes involved in glycolysis, the citric acid cycle, and ATP synthesis. Manganese is a cofactor for enzymes, such as pyruvate carboxylase and isocitrate dehydrogenase, in the citric acid cycle. Copper is a cofactor for cytochrome c oxidase, the last enzyme in the electron transport chain. Zinc is required for the activity of alcohol dehydrogenase and lactate dehydrogenase, which are involved in anaerobic glycolysis [148,150,151].

Metal ions also play a regulatory role in energy metabolism. Calcium, for example, acts as a signaling molecule to activate enzymes, such as pyruvate dehydrogenase, in response to the cell’s energy status. In addition, metal homeostasis is closely linked to energy metabolism, as the uptake, storage, and utilization of metal ions require energy expenditure. A disturbance of metal ion regulation can, therefore, impair cellular bioenergetics and lead to metabolic disorders [149]. 

As already mentioned, divalent metal ions, such as Ni^2+^ and Co^2+^, have significant effects on HIF-1 turnover, and this affects cellular energy metabolism. Upon HIF-1 stabilization, hypoxia-responsive elements (HREs) in the nucleus are activated. This transcriptional activation leads to an upregulation of genes critical for glycolysis, a metabolic pathway that converts glucose into pyruvate, generating ATP in the process. This mechanism is vital under hypoxic conditions, where oxygen availability is limited, inhibiting oxidative phosphorylation, the primary pathway for ATP production in aerobic conditions. This shift to glycolysis provides a rapid source of energy and results in the production of lactate, which can be further utilized by other metabolic pathways or exported from the cell to maintain pH balance. This metabolic adaptation is essential for cell survival and function in low-oxygen environments, highlighting the importance of HIF-1 in cellular energy metabolism. However, metal ion-induced HIF-1 stabilization leads to a paradoxical situation in contrast to hypoxia-induced HIF-1 stabilization, as these supposed “oxygen deficiency adaptation processes” occur in normoxia [152].

Stabilization of HIF-1α by these metal ions often occurs due to their ability to disrupt iron homeostasis by inducing a state of relative iron depletion. This depletion prevents the hydroxylation and degradation of HIF-1α. Regarding cellular energy metabolism, metal ions that activate HIF-1, such as Ni^2+^, Co^2+^, V^5+^, and Mn^2+^, can induce transcription of HIF-1 target genes involved in glycolysis, angiogenesis, and other metabolic pathways. Consequently, this shift towards a more glycolytic, hypoxia-like metabolism can significantly affect cellular energy production and utilization. Conversely, the presence of Fe^2+^ can reverse the induction of HIF-1α by these metal ions, suggesting that iron deficiency is a crucial mechanism by which they affect energy metabolism. Overall, disruption of metal ion homeostasis, particularly with divalent cations, can profoundly affect the regulation of HIF-1 and, consequently, on cellular bioenergetics and energy metabolism.

Through this, metal ions are indispensable cofactors and regulators of the energy metabolic enzymatic machinery that drive energy production and utilization via glycolysis, the citric acid cycle, and oxidative phosphorylation.

### 2.6. Cell Adhesion and Cell Migration

#### 2.6.1. Cell Adhesion and the Extracellular Matrix

Cell adhesion depends on well-orchestrated processes, such as extracellular matrix (ECM)–cell membrane interaction and cytoskeleton-based cell spreading. Several metalloproteins are also involved in these complex processes. Therefore, changes in the balance of metal ions also influence these processes. Physiologically relevant metal cations, such as Mg^2+^, Ca^2+^, and Mn^2+^, are functionally associated with membrane proteins, i.e., the integrins [153,154]. Integrins are responsible for mediating cell adhesion, the cell connection between the ECM and the cytoskeleton. Integrins are integrated into the cell membrane as dimers with an α and a β subunit [155,156]. Integrins exist in distinct conformations: the inactive conformation, with low affinity for ligands, and the active conformation, with high ligand affinity and, thus, high ligand binding capacity [137]. Detailed investigations on the α5β1 integrin revealed three metal ion binding sites essential for the switch to the active conformation, in which the binding of ECM molecules takes place. Under physiological conditions (~1 mM Mg^2+^/~1 mM Ca^2+^), the central metal ion-dependent adhesion site is reversibly occupied by Mg^2+^, while the other two sites reversibly bind Ca^2+^ ions [53]. Fibronectin, the major ligand for the α5β1 integrin, contains an arginine–glycine–aspartate (RGD) motif that connects to the Mg^2+^ ion through its aspartate sidechain. The electron orbitals of the Mg^2+^ ion and the aspartate in the RGD motif overlap, making their bond remarkably strong [157]. 

In 1988, Gailit and Ruosolahti showed that Mn^2+^ can replace Mg^2+^ and Ca^2+^ in the binding sites [52]. It was later demonstrated that the metal ion exchange significantly enhances ECM binding (integrin affinity for the RGD motif was increased ~400-fold) [158]. However, studies have only been performed on isolated integrin ectodomains. A recent study using conformation-specific antibody Fab fragments in intact cells revealed that Mn^2+^ leads to a shift of the conformational equilibrium but does not entirely open integrins. The proportion of integrins in the active conformation is 0.13% under physiological conditions but can be shifted to 4.9% by adding 2 mM Mn^2+^ [153]. This Mn^2+^-induced increased adhesion potential has been proven by cell studies investigating the effect of divalent cations released from different implant materials. In this context, the doping of commonly used implants with divalent cations like Mg^2+^ or Mn^2+^ has not only led to the activation of integrins [154] but also to the increased expression level of integrins in the cells, as shown for Mn-doped ceramics [159], Mn-doped titanium [142], or polyether-ether ketone (PEEK) implants [160,161]. However, these Mn-induced effects are dose-dependent: moderate doses enhance integrin activation, expression, and osteogenic differentiation, while higher levels decrease cell viability and proliferation [162,163]. Due to the integrin-activating and migration-promoting properties of Mn-doped materials, innovative implants have been developed to support tissue regeneration, e.g., for treating chronic wounds [161,164].

Furthermore, integrins are regulated by some cell surface molecules, such as heparan sulfate proteoglycans, and ECM ligands, such as fibronectin and other proteoglycans [158,165,166]. The molecular details of metal ion–ECM interactions are generally much less studied than those of metal–protein interactions. The complex nature of cell membranes makes it challenging to study basic effects, which is why ECM components, such as heparin, are used as a model [167]. An overall trend for heparin–metal affinity and number of binding sites (with highest values for Mn^2+^ and Cu^2+^) has been deduced. However, the micro-heterogeneity of heparin, heparan sulfate, and other ECM-related biomolecules impedes a detailed understanding of their metal ion interactions [168]. In general, metal cations can stabilize sulfate groups against dissociation, which may partly explain the positive effect of these metals on cell adhesion [169].

Although titanium is claimed to be a bioinert metal, it has been shown that the release of titanium ions from implants interferes with the phosphorylation state of focal adhesion kinases (FAKs) through the activation of ROS-dependent pathways, finally increasing the adhesion capability of pre-osteoblasts [170]. Other metal ions putatively released from implants, such as ions from CoCr implants (nanomolar range), induced β1-integrin gene activation and affected integrin-based downstream signaling [171]. In contrast, the exposure of endothelial cells in vitro to Co^2+^ ions led to an initial impairment of cell adhesion, whereby a connection with the activation of integrins was also suspected here [172]. Experiments on CoCr implant surfaces revealed significantly decreased cell adhesion and size of cellular adhesion contacts compared to titanium or tissue culture polystyrene surfaces of the same roughness [173]. These effects may be guided by the induction of inflammatory activation (Section 2.4) and Co^2+^-induced hypoxia signaling (Section 2.5). 

#### 2.6.2. Cell Migration and the Cytoskeleton

Cell migration is the process by which cells move from one location to another, and its tight regulation is crucial for maintaining tissue homeostasis. Cell migration includes both cell adhesion and detachment [174]. Deregulation of migration can lead to pathophysiological conditions, such as those observed in the spread of tumors in the body (metastasis) [175]. The cellular migration machinery comprises the cytoskeleton, the plasma membrane, and the proteins therein. Migration is facilitated by the polarization of cells along the axis of movement, and this polarization is manifested in the formation of a lamellipodium via the polymerization of actin filaments at the front of the cell and the retraction of the rear part, which is controlled by contractile proteins [176]. This asymmetry requires an asymmetric distribution of adhesion receptors, the integrins, in migrating cells [177]. For cell migration, the distinct disassembly of cell–matrix adhesions at the rear of the migrating cell is of eminent importance and is regulated by calcium-sensitive proteases. The local Ca^2+^ gradient, with increased Ca^2+^ concentrations in the cell´s rear part, supports this disassembly [178]. D’Souza et al. found evidence that distinct calcium channels cluster near cell–matrix adhesions, essential for adhesion disassembly [179]. 

In general, other divalent cations also influence cell migration in a relevant manner. Modulating the extracellular concentrations of Mg^2+^, Ca^2+^, Zn^2+^, and Mn^2+^ may stimulate or inhibit migration. Melanoma cells, for instance, migrate more efficiently on a collagen IV substrate when exposed to higher concentrations of Mg^2+^, while Ca^2+^ does not affect such activity. On the other hand, both cations are relevant for migration on fibronectin substrates [180]. Another metal ion-dependent enzyme family is the matrix metalloproteinases (MMPs), with Zn^2+^ as the responsible ion, which enables the cleavage of various ECM molecules. With this ability, MMPs enable remodeling during various physiological processes, such as blood vessel formation and many further aspects of tissue regeneration [168]. MMPs are involved in cell migration by degrading ECM molecules [169].

Cell shape and migration depend highly on the dynamics in the intracellular filaments like actin fibers or intermediate filaments. The bundling and unbundling of the fibers enable controlled cell motility [176]. For example, bundling and crosslinking of actin networks occur by divalent counter ions, such as Mg^2+^ forming cross-bridges between actin filaments. Recent research has proven that only modest bundling and network rearrangement by altering Mg concentration is required to induce dramatic changes in the elasticity and stiffness of the networks [181]. Multivalent cations differ in their effectiveness in bundling/aggregating the intermediate filament vimentin, making transition metal ions more efficient [182]. However, it has to be considered that intracellular concentrations are tightly regulated, and it has to be elucidated whether such cross-bridging events occur in the cellular environment or may only be relevant in pathological situations to study the mechanisms of metal toxicity [183]. Further observations indicate that maintaining the integrity of the F-actin cytoskeleton is necessary for the appropriate intracellular location of the endoplasmic reticulum and Ca^2+^ dynamics [48]. However, the inhibition of microtubule formation had no inhibitory effect on Ca^2+^ influx, suggesting that microtubules are not required to active calcium [184].

#### 2.6.3. Primary Cilia

Primary cilia are non-motile, membrane-bound organelles found in most types of eukaryotic cells. They serve primarily as sensory organelles to coordinate various signaling pathways crucial for cellular homeostasis and development [185]. Primary cilia protrude from the surface, and their function is based on a dynamic, tissue-specific cycle of assembly and disassembly. The core structure of the primary cilium is commonly referred to as the ciliary axoneme and consists of microtubules arranged circumferentially along its longitudinal axis [186]. Primary cilia are crucial for mechanotransduction and other signaling pathways, activating molecular mechanisms that depend on Ca^2+^ influx through channels along the ciliary axoneme. A comparable calcium flux has also been observed in osteoblasts and osteocytes in response to flow and suggests that primary cilia hold a comparable mechanosensory role in bone as in other tissues [187]. 

As previously described, metal ions influence the formation and interconnection of the cytoskeleton, which also influences the development and stability of the primary cilium. It was shown that non-toxic levels of cobalt ions induced histone deacetylase (HDAC6)-dependent primary cilia disassembly in chondrocytes, which was associated with α-tubulin deacetylation leading to primary cilia shortening [188]. This shortening can affect the function of the primary cilia, such as mechanotransduction [188,189]. However, others report an increase in the length of primary cilia after exposure to non-toxic Co^2+^ concentrations [190]. Thus, any variation in the experimental conditions may lead to different results, indicating the current uncertainty of the effects.

### 2.7. Blood Vessel Formation

The blood vessels constitute the body’s circulatory system, transporting blood through and enabling the exchange of oxygen, nutrients, waste products, and cells, or biologically active components (e.g., immune cells, antibodies, and much more). Therefore, the process of new blood vessel formation is also crucial for individual growth and regeneration, e.g., after injuries [191]. The conditions that influence the formation of new blood vessels and the behavior of MSCs are mutually dependent. Thus, the altered microenvironment created by the formation of new blood vessels is reflected in the behavior of MSCs, as well as the fact that MSCs release factors (so-called pro-angiogenic factors) that support the formation of new blood vessels e.g., in hypoxia or hypoxia-related signaling. These MSC-derived pro-angiogenic factors include, for example, vascular endothelial growth factor (VEGF), which promotes the migration and proliferation of endothelial cells, processes necessary for blood vessel formation. Specific signaling systems of pro-angiogenic factors control each step of blood vessel formation [192]. MSCs can also promote the differentiation of other cells into endothelial cells during inflammation or tissue damage [193]. Therefore, the formation of new blood vessels influences MSCs and vice versa. 

As metal ions are critical to numerous biological processes, their balance is also crucial for properly forming and maintaining blood vessels. Ca^2+^ signaling drives new vessel formation by recruiting multiple Ca^2+^-sensitive decoders in response to signals, such as VEGF or basic fibroblast growth factor (FGF-2), in endothelial cells. Furthermore, intracellular Ca^2+^ signaling stimulates proliferation, tube formation, and new vessel formation in endothelial progenitor cells [194]. Ca^2+^ signaling involves complex interactions within the cells involved, which are regulated by different signaling pathways, such as specific proteins and channels and non-specific metal ions, such as Zn^2+^, Ni^2+^, and Co^2+^, which may compete with Ca^2+^ for binding sites on calcium-dependent proteins, enzymes, and channels (Section 2.2). This competition could alter normal signaling pathways and disrupt the role of calcium signaling in blood vessel formation. Furthermore, metal ion-induced increased oxidative stress may affect blood vessel formation and remodeling (Section 2.3). 

A further important step in blood vessel formation is the specific dissolution of the ECM and migration of the involved cell populations (i.e., endothelial cells, pericytes, progenitor cells, etc.). Therefore, the activity of MMPs with a Zn^2+^ in the catalytic domain is decisive as it participates directly in the cleavage of peptide bonds within the ECM molecule [195]. Zn^2+^ in the catalytic domain can be substituted by other metal ions, like Cu^2+^, Co^2+^, Mg^2+^, and Mn^2+^, which affects MMP activities depending on the specific metal ion involved [196,197,198,199]. Variations in metal ion availability can influence MMP activity and, thus, cell migration in different tissues and pathological conditions. This can affect processes, such as tissue remodeling and wound healing, and the progression of diseases, like cancer, arthritis, and fibrosis [200].

However, Cu^2+^ has also been shown to affect the formation of blood vessels, primarily through its involvement in lysyl oxidase activity. Lysyl oxidase catalyzes the oxidative deamination of lysine residues in collagens and elastins, which leads to the formation of cross-links that are essential for the integrity and elasticity of the ECM and, thus, for the integrity of blood vessels [201]. Cu^2+^ also plays an essential role in the function of angiogenin, a protein that promotes the formation of blood vessels. The interaction of Cu^2+^ and angiogenin enhances the binding of angiogenin to endothelial cells, thereby stimulating blood vessel formation [202]. Additionally, Cu^2+^ regulates fibroblast growth factor-1 (FGF-1) release, further promoting blood vessel formation [203]. In this case, there are indications of interference by a non-specific metal ion with Cu^2+^. For example, the activity of lysyl oxidase is significantly reduced by increased Zn^2+^ concentrations [204]. However, Zn^2+^ has also been shown to affect the angiogenin binding to endothelial cells [205]. Nevertheless, unlike previously, other non-specific metal ions, like Ni^2+^ and Co^2+^, do not appear to disturb the Cu^2+^ interactions described for lysyl oxidase, angiogenin, and the FGF-1 protein complexes. This suggests a degree of specificity in the metal ion interactions with Cu^2+^ and Zn^2+^.

HIF signaling is an outstanding signaling pathway for forming new blood vessels, and as described earlier, metal ions, such as Co^2+^, have an apparent stimulatory effect in this signaling pathway [76,152,172] (Section 2.5). Therefore, developing specific biomaterials, such as Co^2+^-doped bioactive glasses, which could accelerate wound healing by triggering the formation of blood vessels through their influence on the HIF signaling pathway, is of great clinical interest [80].

Since Cu^2+^ also stimulates the process of blood vessel formation by mechanisms other than Co^2+^, the combination of Cu^2+^ and Co^2+^ could synergistically stimulate blood vessel formation, requiring lower individual doses compared to their individual effects [76]. These ions could synergistically enhance the angiogenic potential of MSCs and, thus, promote faster and more effective blood vessel formation. However, this approach also carries risks, as increased or unbalanced metal ion concentrations could lead to altered cell signaling, oxidative stress, and inflammation. Therefore, optimizing dosing and delivery methods is crucial to maximize the therapeutic benefit while minimizing side effects. An illustrative example of the complexity of the metal-induced effects was demonstrated in an animal model. Here, exposure to CoCrMo alloy debris in the dorsal skinfold chamber of hamsters resulted in a complete collapse of the microvasculature, which was not the case with debris from various titanium–aluminum alloys [206] and was not necessarily to be expected due to the effects described for Co^2+^.

To summarize, maintaining an appropriate homeostasis and balance of metal ions, even beyond the regulation of Zn^2+^, Co^2+^, and Cu^2+^, is beneficial for blood vessel formation and vascular integrity. Disturbances, such as an overload or an imbalance of metal ions, can have a detrimental effect.

## 3. Discussion

MSCs are an essential component of tissue regeneration because they differentiate into various cell types, such as osteoblasts, chondrocytes, and adipocytes. However, the functions of MSCs (including self-renewal and differentiation) can be significantly modulated by the presence of metal ions. Altered differentiation or the specific absence of differentiation of MSCs can be essential for any regenerative process and tissue homeostasis [195]. Furthermore, achieving a certain differentiation status may be crucial for the successful cell therapeutic application of MSCs. However, many signaling pathways required for targeted differentiation of MSCs still need to be completed. Therefore, it is of considerable interest to expand the knowledge about the effects of metal ions on the behavior of MSCs.

**Figure 2:**
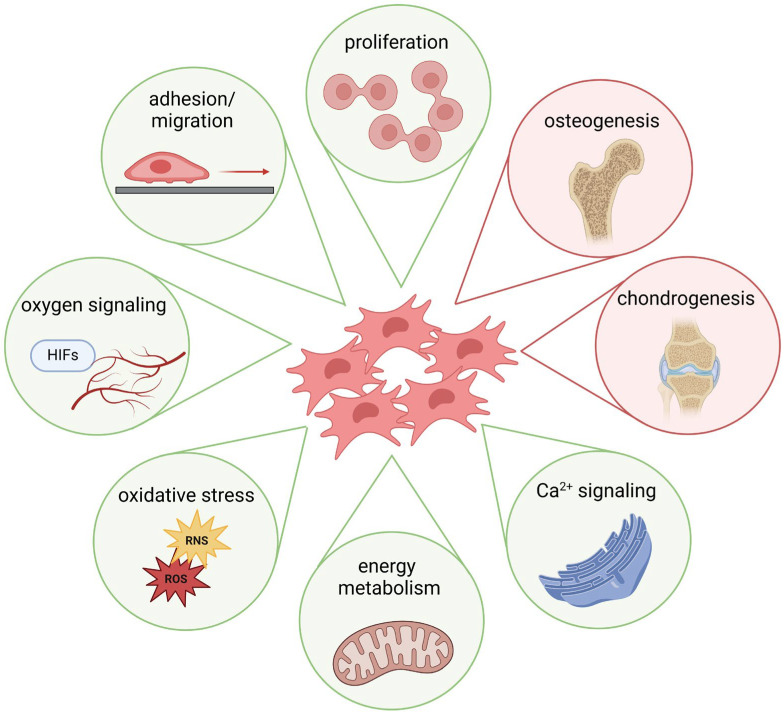
Schematic depiction of exemplary metal ion-induced effects on MSC function and differentiation (created with BioRender.com, agreement No. AD274QKS6F).

The previous sections demonstrated that metal ions, starting from their cellular uptake, influence various cellular signaling processes and, thus, the differentiation of MSCs (Figure 2). Metal ions, such as Ca^2+^, have been shown to promote MSC osteogenic differentiation via multiple pathways, including activation of the mitogen-activated protein kinase (MAPK) signaling pathway and the Wnt/β-catenin signaling pathway, both of which are critical for bone formation [207]. Furthermore, increased extracellular Ca^2+^ administration has been shown to promote MSC proliferation, migration, and matrix mineralization [208]. In addition, contact with calcium phosphate materials promotes osteogenic differentiation of MSCs [209]. Zn^2+^ also promotes osteogenic differentiation in human MSCs by activating different osteogenesis-related signaling pathways [210,211]. Moreover, Mg^2+^ can activate the Notch1 signaling in MSCs, which can influence the proliferation and differentiation of osteoblasts, as reflected by an increase in mineralization [117,212]. Cu^2+^ has also been shown to affect MSC proliferation and differentiation positively [213]. However, little is known about the effects of a disturbed metal ion balance on osteogenesis: Li et al. described a mechanism for decreased mineralized matrix deposition by high amounts of Mg^2+^ via suppressing mitochondrial Ca^2+^ concentration and autophagy [214]. Schrock et al. demonstrated that Co^2+^ affected the osteogenic differentiation of MSCs, likely through hypoxia signaling [215]. The benefits of metal ion-induced hypoxia signaling (e.g., by Co^2+^) are mainly considered through an indirect gain due to a better supply of blood vessels and, thus, nutrients and growth factors. Interestingly, the effects of “true” hypoxia, i.e., an O_2_ concentration of 1%, were variable; while some studies describe a loss of differentiation potential [216,217], others showed an increase in differentiation of human MSCs [218].

Metal ion-induced oxidative stress could also be one of the factors responsible for the altered differentiation capacity of MSCs. MSCs play a crucial role in coping with oxidative stress through various mechanisms [106], but the differentiation potential of MSCs is significantly impaired by treatment with higher amounts of ROS [219,220]. The effects of oxidative stress caused by metal implants are still poorly understood in detail, but efforts are being made to equip implants with antioxidant properties to combat this problem [221].

The influence of metal ions on the chondrogenic differentiation of MSCs is both poorly researched and also characterized by heterogeneous data. While some metal ions, such as Fe^2+^, Mg^2+^, and Co^2+^, have been investigated for the chondrogenic differentiation of MSCs, there is little specific information on the effects of other metal ions, such as Ni^2+^. For example, there is evidence of inhibition of chondrogenic differentiation of human MSCs upon exposure to iron oxide [222], while others found promotion of chondrogenic differentiation in rat MSCs [223]. Exposure of MSCs to CoCl_2_ also showed divergent effects; whereas CoCl_2_ did not induce chondrogenic differentiation in MSCs from human bone marrow [224], it led to increased expression of chondrogenic markers in a murine MSC line [225]. Since stimuli that increase intracellular Ca^2+^ can influence the chondrogenic differentiation of MSCs [226,227], the metal ions that affect Ca^2+^ levels should also affect MSC differentiation.

There is very limited data on metal ions’ effects on adipogenic MSC differentiation. Most studies focus on other aspects of MSC differentiation (e.g., oxidative stress and osteogenic differentiation). In a 2022 study, doping polyelectrolyte multilayers of chitosan and alginate with Cu^2+^ and Fe^3+^ led to the promotion of adipogenesis of multipotent mouse cells (i.e., C3H10T1/2 fibroblasts), whereas doping with Ca^2+^ and Co^2+^ did not lead to significant adipogenic differentiation [228]. Thus, more studies, systematic reviews, or meta-analyses are needed on the effects of particularly sub-toxic concentrations of metal ions on adipogenic differentiation. This emphasizes the need for further systematic research to gain a more comprehensive and consistent understanding of the effects on adipogenic differentiation of MSCs.

An important aspect to consider is the formation of ROS triggered by metal ions, which can lead to oxidative stress and potentially impact cellular senescence. Senescent cells are often characterized by a secretory phenotype involving the release of inflammatory and proteolytic proteins [229]. Since stress-induced and replicative senescence are mutually reinforcing processes [230], these effects may contribute to loosening metallic endoprosthetic implants due to corrosion products [231]. Similar stress-induced effects on cellular aging have also been observed in MSCs; elevated ROS levels accelerate senescence, promote adipogenic differentiation, reduce osteogenic differentiation, and impair the immunomodulatory activity of MSCs [98,232,233]. The appropriate functioning of these processes is crucial for the clinical application of MSCs.

To summarize, the effects of metal ions are strongly influenced by different factors, such as concentration, duration of exposure, and specific environmental conditions, and are, therefore, context dependent. Different cell sources and detection methods also contribute to the heterogeneity of the data. These considerations emphasize the need for further systematic research to gain a more comprehensive and consistent understanding of the effects of metal ions on MSC differentiation.

## 4. Conclusions and Future Directions

Metal ions play a complex and multifaceted role in the differentiation of MSCs, the effects of which are highly dependent on concentration, exposure duration, and microenvironmental conditions; these conditions still need to be better understood. The interplay between metal ions and MSCs has significant implications for the development of biomaterials, implant design, and regenerative medicine strategies. Key findings suggest that metal ions can modulate the behavior of MSCs via several mechanisms, including the activation of HIF signaling pathways. The HIF signaling pathway is critical for cellular adaptation to oxygen availability and regulates fundamental processes, such as metabolism, energy production, and vascular function. The ability of specific metal ions to influence HIF signaling pathways opens up new possibilities for controlling the fate and function of MSCs. 

Furthermore, metal ions can catalyze the formation of ROS through Fenton or Haber–Weiss reactions, leading to increased oxidative stress in MSCs. This oxidative stress can impair the function and differentiation of MSCs. Elevated ROS levels can, for example, inhibit osteogenic differentiation or induce cell death in the case of excessive oxidative stress. However, MSCs also have adaptive mechanisms to deal with moderate levels of oxidative stress.

Future research directions should focus on elucidating the detailed mechanisms by which different metal ions influence the differentiation of MSCs, with particular attention to dose–response relationships and temporal dynamics. In addition, the interplay between metal ions and other factors that influence MSC behavior, such as mechanical forces and the interplay between different growth factors and ECM components, should be investigated. In addition, advanced biomaterials and implants that exploit metal ion-induced effects could be developed to enhance MSC-mediated tissue regeneration while minimizing potential negative consequences, such as oxidative stress. Exploring the therapeutic potential of metal ion-based approaches could mean targeted modulation of MSC function in various clinical contexts, including bone and cartilage repair, cardiovascular regeneration, and immunomodulation.

By addressing these research areas, we can gain a more comprehensive understanding of the metal ion-induced effects on MSC differentiation. This knowledge will help to increase the safety of implants and improve the clinical use of MSCs in regenerative medicine, leading to innovative therapeutic strategies that exploit the potential of metal ion-MSC interactions.
